# Prefabricated Zirconia Crowns and Preformed Metal Crowns in the Treatment of Severely Childhood Caries and Anterior Crossbite in a Child with Autistic Spectrum Disorder

**DOI:** 10.1155/2024/5556502

**Published:** 2024-02-15

**Authors:** Shuman Deng, Qi Gao, Li Zhang, Jing Xie, Ying Chen, Xuezhen Peng

**Affiliations:** ^1^Shenzhen Children's Hospital of China Medical University (CMU), Shenzhen 518026, China; ^2^Department of Stomatology, Shenzhen Children's Hospital, Shenzhen, Guangdong 518026, China; ^3^Department of Maxillofacial Surgery, Shenzhen Second People's Hospital, Shenzhen, Guangdong 518029, China

## Abstract

Crowns have been recommended to treat decayed teeth and rebuild teeth function. The dental management of children with autism is a tremendous challenge for pediatric dentists due to the impaired behaviors and communication disorders. In this context, a 5-year-old boy with autism was treated to solve carious lesions under the assistance of general anesthesia. The posterior occlusal function was restored, and the crossbite existing in the primary anterior teeth was approached merely by NuSmile® zirconia crowns (ZCs) rather than orthodontic intervention. We conducted an 18-month period. Throughout the long-term follow-up, the boy's masticatory efficiency was remarkably improved and the anterior teeth had transferred into the correct position with adequate overbite to maintain the new relationship, thus ameliorating the appearance of tissue on the labial surface and enhancing his quality of life and oral health.

## 1. Introduction

Dental caries is reckoned as the most common chronic infectious disease among children. Untreated dental caries may endanger dental pulp tissues [1]. When decay invades dental pulp, pulpectomy is the reference treatment for vital primary molars with irreversible pulpitis [2]. The case studies suggest that tampon-based coronal pulpotomy using a calcium silicate–based biomaterial such as iRoot BP (Innovative BioCeramix, Vancouver, BC) can be a reliable approach for the treatment of such teeth. Although a pulpless tooth can remain functional in the oral cavity, pediatric dentists still advocate regenerative approaches for primary teeth [3].

Autism or autistic spectrum disorder (ASD) is defined as a severe developmental disorder characterized by the classic triad of impaired communication, socialization, and repetitive and restricted patterns of behavior [4]. Disabled children are usually uncooperative with dentists to fulfill consultation, examination, and treatment owing to their congenital intelligence retardation and language development deficits [5]. Although the incidence of dental caries in children with ASD is not significantly higher than that in normal children, once the children have tooth decay, they often leave them untreated timely and effectively as we expected. Therefore, oral comfort treatment is of paramount importance. Treatment under general anesthesia (GA) can solve all the oral problems at a time, reduce the frequency of visits, and curtail the health care costs. Meanwhile, the procedure has been affirmed and recognized worldwide [6].

The term anterior crossbite refers to an abnormal labiolingual relationship between one or more maxillary and mandibular incisor teeth. This may be termed a reverse overjet when the patient is in centric occlusion and one or more maxillary incisors are positioned lingually to the mandibular incisor teeth. Anterior crossbite has an incidence of 4-5% approximately and usually exaggerates during mixed dentition [7]. A variety of methods have been proposed to correct anterior crossbite, such as tongue blades, reversed stainless steel crowns, fixed acrylic planes, bonded resin-composite slopes, and removable acrylic appliances with finger springs [8], but these applicances beforementioned are hardly worn in children with severe behavior disorders as well as with intellectual or physical disabilities.

Prosthodontic treatment is broadly used in decayed primary teeth, and sorts of techniques such as polycarbonate-formed crowns, resin clear-formed crowns, and metal stainless steel crowns (SSCs) have been boomed in recent decades [9–11]. Recently, the aesthetic restoration gained more attention with an increasingly widespread application due to soaring aesthetic requirements, which is a challenge to pediatric dentists especially in very young children or children with any disability. NuSmile® zirconia crowns (ZCs), a brand of prefabricated ceramic crown, has been extensively used to restore decayed primary teeth. Compared to SSC or ZCs, composite strip crowns are predisposed to have adverse consequences in some aspects, such as retention, durability, marginal seal, and color. With delicate nature replicating aesthetics, ultimate durability, and convenient placement, ZCs have become a silver bullet [12].

The aim of the current study is to use the zirconia crowns and preformed metal crowns to cure the decayed primary teeth and crossbite malocclusion under general anesthesia in a 5-year-old autistic child.

## 2. Case Report

We received a 5-year-old autistic boy with widely decayed teeth and maxillary anterior crossbite. The child had no intelligible speech and was totally uncooperative in the dental chair (Dentsply Sirona Inc, Pennsylvania State, Germany).

The whole oral examinations and procedures were performed by a senior associate chief physician. Percussion testing was performed by tapping the buccal tooth surface with a hard instrument—such as the opposite end of a dental mirror (Hedva, Guangdong, China)—to test the clinical status of the periodontal ligament (PDL) [13]. Palpation examination was used to test the sensitivity of the gingival tissue with the examiner's hand [14]. Tooth mobility was probed by oral tweezers and probes (Hedva, Guangdong, China) [15].

**Figure 1:**
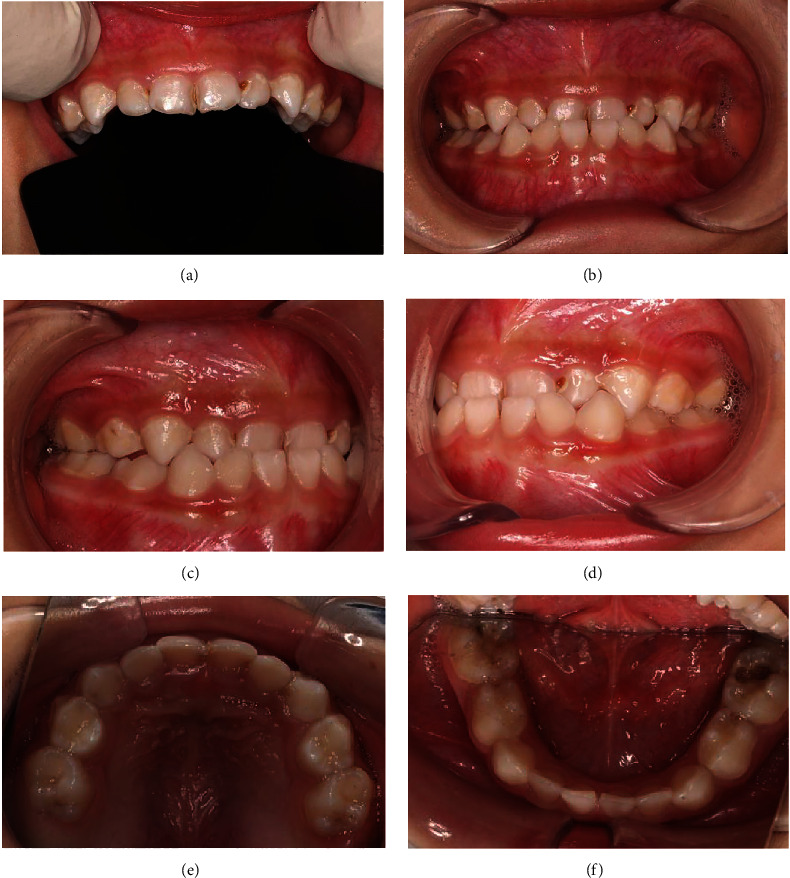
Intraoral photos of the patient before treatment: maxillary anterior teeth (a), frontal occlusion (b), right lateral occlusion (c), left lateral occlusion (d), upper occlusion (e), and lower occlusion (f).

**Figure 2:**
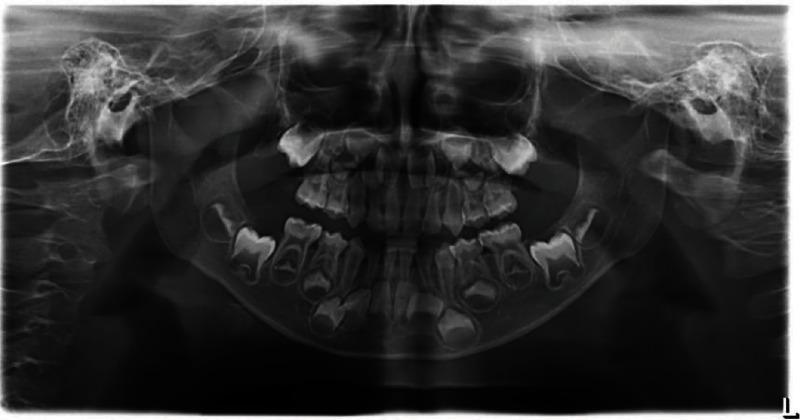
Panoramic X-ray of a 5-year-old patient.

Oral examination revealed multiple teeth with deep occlusal/proximal caries but normal surrounding soft tissue (Figure 1). Percussion/palpation tests showed neither sensitivity nor mobility of the teeth. A preoperative radiograph (Sirona Orthophos XG 3D, Bentzheim, Germany) confirmed normal continuous periodontium and deep carious lesions or involving the pulps (Figure 2).

Given that the patient presented with no pain or discomfort, absence of spontaneous pain, no response to percussion/palpation, and radiographic examinations reveals normal periapical situations, the initial diagnosis was deep caries or asymptomatic pulpitis [16]. After perfecting the preoperative examination and preparation, no obvious contraindications were found, and the treatment plan was established followed by thorough communication with his parents and then signed informed consent. We finally chose to treat under the assistance of anesthesia machine AX-500 (ICEN, Guangdong, China).

**Figure 3:**
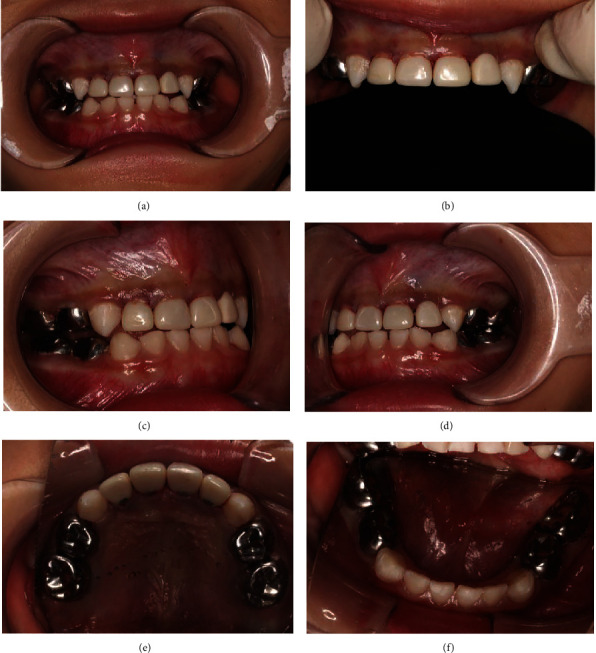
Postoperative result. Oral surgery was performed under general anesthesia: frontal occlusion (a), maxillary anterior teeth (b), right lateral occlusion (c), left lateral occlusion (d), upper occlusion (e), and lower occlusion (f).

The child was placed in the supine position and routinely disinfected and toweled after successful induction of general anesthesia and tracheotomy tube (Wellead Medical Co., Ltd, Guangzhou, China). Local anesthesia was placed with 4% articaine-added 1 : 100000 epinephrine (Produits Dentaires Pierre Rolland, Merignac, France). One of three treatments can be selected: direct pulp capping (where a healing agent is placed directly over the exposed pulp), pulpotomy (removal of a portion of the pulp), or pulpectomy (removal of the whole pulp in the pulp chamber and root canal of a tooth) [17]. The tooth was directly filled with Filtek™ Z350 XT (3M ESPE St. Paul, MN, USA, shade A2) [18] or performed vital pulpotomy using a pulp-capping agent iRoot BP (Innovative BioCeramix, Vancouver, BC) [19], depending on the pulp vitality. If vital pulpotomy was selected, the roofs of the pulp chambers were removed completely, and the pulps were amputated to the root orifice level with a round diamond bur BR-31 (MANI, Inc., Utsunomiya, Japan). Hemostasis was obtained by abundant irrigation with 0.9% sterile normal saline (Kelun, Sichuan, China) and 1% NaClO (Langli, Wuhan, China) repeatedly and alternatively. A 2-millimeter layer of iRoot BP Plus (Innovative BioCeramix, Vancouver, BC) was pressed on a pulp surface gently. The teeth were then built up with core materials (GlasIonomer FX Ultra, SHOFU INC, Japan) [20, 21]. Finally, the molars were restored with preformed metal crowns [22] (Shinhung Co., Ltd., Jung-gu, Seoul, Korea), and prefabricated zirconia crowns [23](NuSmile®, Houston, TX, USA) were applied to the maxillary anterior teeth. Immediate postoperative images were taken by Nikon D750 (Nikon Corporation, Japan) for documentation and scientific research (Figure 3). Follow-up examinations were scheduled to be performed at 3 and 6 months postoperatively and every 6 months thereafter.

**Figure 4:**
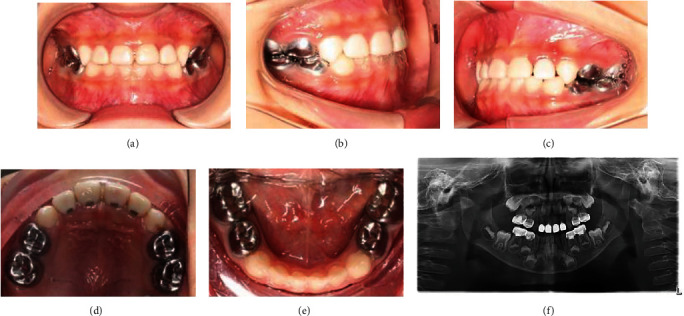
6 mon recall examinations. All restorations in situ, no abnormalities. Note the presence of occlusal reconstruction: frontal occlusion (a), right lateral occlusion (b), left lateral occlusion (c), upper occlusion (d), lower occlusion (e), and panoramic X-ray (f).

**Figure 5:**
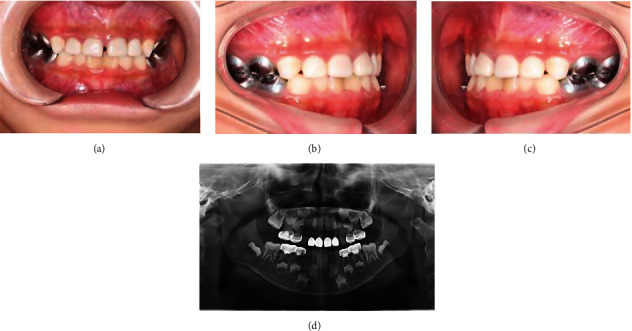
18 mon recall examinations. All restorations in situ, no abnormalities. Note the presence of occlusal reconstruction: frontal occlusion (a), right lateral occlusion (b), left lateral occlusion (c), and panoramic X-ray (d).

By self-regulation of the occlusal relationship, an anterior crossbite was successfully transmitted to a normal bite over a 6-month period (Figure 4). During the 18-month recall period, the treated teeth showed normal clinic outcomes and radiographic signs (Figure 5), and the crowns had manifested good retention and aesthetic results.

## 3. Discussion

In our study, we used preformed metal crowns to restore the posterior occlusal function and zirconia crowns to solve the anterior crossbite in a 5-year-old autistic boy without orthodontic intervention. After an 18-month follow-up, his masticatory function was ameliorated, and his crossbite was correctly addressed, thus improving the oral environment and providing pleasing facial aesthetics, so as the psychosocial development. As the patient was completely uncooperative, extra- and intraoral photos were taken at no time but 6 and 18 months after treatment. However, gaps between the maxillary anterior teeth were observed 6 months after treatment, probably due to the excessive stress in his anterior area.

The American Academy of Pediatric Dentistry (AAPD) recognizes that individuals with ASD are prone to increase the opportunity of calculus formation, gingivitis, periodontitis, enamel hypoplasia, dental caries, anomalous formation of teeth, and malocclusion. This report demonstrated that ASD children were more likely to develop class III occlusion, which was consistent with other studies [24, 25].

An incisor eruption usually leads to a crossbite relationship in centric occlusion, and early orthodontic treatment is indeed with the least delay possible. Individual treatment could be performed to the types of class III malocclusions such as rapid maxillary expansion, maxillary protraction, and chin cup in skeletal class III malocclusions and removable appliance and inclined guide plate in pseudo class III malocclusions [26–28].

In our case, the autistic child had multiple tooth decay and crossbite as well. After endodontic treatment under general anesthesia, zirconia crowns and preformed metal crowns were chosen to restore the affected teeth, solving the aesthetic problem of the anterior teeth while correcting the crossbite. Occlusal elevation was inevitable with preformed metal crown restorations, especially when multiple primary molars were restored simultaneously. It was found that the occlusal vertical distance returned to its primary position within 1 month after preformed metal crown restoration [29], and the results were consistent with this case. The mechanism is linked with the rapid growth of alveolar bone and the strong adaptability of condyles in children [30].

Zirconia crowns and preformed metal crowns can not only restore the occlusal relationship and chewing function but also preserve the anatomical shape of teeth. However, it requires proficient clinical skills, and the medical costs are high. Therefore, some doctors still use resin filling methods to repair tooth decay and correct the crossbite, achieving good clinical outcomes.

Further evidence was confirmed in this report that children with ASD were willing to perform treatment under general anesthesia due to uncooperation in a dental setting [31].

Early childhood caries is characterized by early onset, rapid progression, and easy involvement of the pulp, and such children are unable to cooperate with complicated carious treatment due to their young age and autism. Different categories of glass ionomer cement and composite resin materials have been recognized as the preferred choices. However, they have cons and pros. Complications such as the part or the whole shedding of filling materials and microleakage often occur. Advancements in dental restorative techniques have occurred over the past decade. The AAPD guidelines state that preformed metal crowns (PMCs) are highly recommended for all primary molars in children under general anesthesia to lower the risk of caries [32]. Stainless steel crowns have a huge advantage of easy placement and durability outperforming other filling materials [33]. Overall survival analysis showed PMCs to be significantly more successful than restorations, particularly in uncooperative children [34].

However, PMCs have a nonaesthetic appearance and become a point of concern. Recently, prefabricated zirconia crown (PZC) has been gradually used as an aesthetic restorative material for primary teeth [35]. To some other doctors, a composite strip crown is an ideal choice [36]. Compared to composite strip crowns, zirconia crowns show better gingival health, less plaque accumulation, and excellent biocompatibility [37].

Additionally, in combination with therapies, prevention strategies should be evaluated. Historically, fluoride has been the first attempt in dental practice used for preventive purposes [38]. Recently, casein phosphopeptide-amorphous calcium phosphate [39] and biomimetic hydroxyapatite [40] have been introduced and have shown promising results. These products should be tested in future trials and also in patients with low compliance levels.

In the present study, there are some limitations that we need to improve. Although caries is a common disease, making an accurate diagnosis of the precise disease can be a huge challenge for even the most skilled dental practitioner. The pulp, its depth/extent, activity, and the restorability of the tooth should be also taken into account. In order to develop the most appropriate treatment strategy for the patient, the clinician should obtain thorough information from the patient's history (symptomology, diet, oral hygiene, toothbrush, tooth floss etc.), visual inspection, optimal radiographs, and other tools such as CarieScan, quantitative light-induced fluorescence (QLF), DIAGNO laser system, polarization-sensitive optical coherence tomography (OCT), frequency-domain infrared photothermal radiometry (PTR) and modulated luminescence, species-specific monoclonal antibodies, and artificial intelligence [41].

## 4. Conclusions

This study revealed that children with ASD may face many barriers in accessing oral health care and exhibit more unmet needs for dental treatment. Zirconia crowns may be a reliable means for the correction of crossbite in the primary anterior teeth and open a new orthodontic pathway. The method is simple to perform, changing the patient's passive acceptance to active proficiency. It is easy to control the orthodontic force, with a shorter treatment course, few follow-up visits, more comfort, and ideal aesthetics. These promising initial findings should be followed by further trials. Furthermore, the depth of a carious lesion can be approximately evaluated through a bitewing radiograph. A more accurate impression can be depicted on a cone-beam computed tomography (CBCT); however, this has limitations such as high radiation dose, long shooting time, and image distortion due to uncooperation.

## Abbreviations

ZCs: Zirconia crowns
ASD: Autistic spectrum disorder
PMC: Prefabricated metal crown.
